# Hyperthermic intraperitoneal chemotherapy following up-front cytoreductive surgery versus cytoreductive surgery alone for isolated synchronous colorectal peritoneal metastases: A retrospective, observational study

**DOI:** 10.3389/fonc.2022.959514

**Published:** 2022-10-18

**Authors:** Xiusen Qin, Mohamed Siyad Mohamed, Yuanxin Zhang, Yuefang Chen, Zhijie Wu, Rui Luo, Liang Yi, Hui Wang, Huaiming Wang

**Affiliations:** ^1^ Department of Colorectal Surgery, The Sixth Affiliated Hospital, Sun Yat-sen University, Guangzhou, China; ^2^ Guangdong Institute of Gastroenterology, Guangdong Provincial Key Laboratory of Colorectal and Pelvic Floor Diseases, Supported by National Key Clinical Discipline, The Sixth Affiliated Hospital, Sun Yat-sen University, Guangzhou, China; ^3^ Department of Anesthesia, The Sixth Affiliated Hospital, Sun Yat-sen University, Guangzhou, China; ^4^ Department of Anorectal Surgery, Liangzhou Hospital, Wuwei, China

**Keywords:** colorectal cancer, synchronous peritoneal metastases, cytoreductive surgery (CRS), hyperthermic intraperitoneal chemotherapy (HIPEC), prognosis

## Abstract

**Background:**

To date, the value of hyperthermic intraperitoneal chemotherapy (HIPEC) following up-front resection for isolated synchronous colorectal peritoneal metastases seems controversial.

**Patients and Methods:**

This retrospective cohort study was conducted from September 1, 2012, to September 1, 2019, at a tertiary medical center in China. Patients with isolated synchronous colorectal peritoneal metastases were included in CRS plus HIPEC group or CRS alone group based on the treatment history. Overall survival and relapse-free survival were estimated using Cox proportional hazards regression analysis and Kaplan–Meier method.

**Results:**

78 patients with isolated synchronous colorectal peritoneal metastases were identified among 396 patients with synchronous colorectal peritoneal metastases. 43 were in the cytoreductive surgery plus HIPEC group and 35 were in the cytoreductive surgery alone group. Among them, 61 patients had relapse-free survival data. The median peritoneal cancer index was 4 in all patients. After a median follow-up of 46.0 months, 5-year overall survival was 66.8% and the median relapse-free survival was 36.0 (95% CI, 6.8-65.1) months in the CRS plus HIPEC group. 5-year overall survival was 31.2% and the median relapse-free survival was 12.0 (95% CI, 9.0-15.0) months in the CRS alone group. Cox regression analyses showed that HIPEC was the independent prognostic factor for overall survival (*P* = 0.004) and relapse-free survival (*P* = 0.049).

**Conclusion:**

Findings of the present study suggest that HIPEC following up-front CRS could improve overall survival and relapse-free survival in patients with isolated synchronous colorectal peritoneal metastases.

## Introduction

Colorectal cancer (CRC) is the third most common cancer and one of the most common causes of cancer-related death worldwide (1). The peritoneum is the second most frequent site of colorectal cancer metastases (2). Approximately 7% of patients with CRC will be diagnosed with peritoneal metastases at the time of initial diagnosis, which is defined as synchronous peritoneal metastases (3). Patients with colorectal peritoneal metastases traditionally were treated with systemic chemotherapy only, with a median overall survival (OS) of less than 12 months (4).

In the last two decades, numerous studies suggested that cytoreductive surgery (CRS) plus hyperthermic intraperitoneal chemotherapy (HIPEC) could improve survival outcomes in CRC patients with peritoneal metastases (4–7). In the Netherlands, patients with isolated synchronous colorectal peritoneal metastases routinely undergo CRS plus HIPEC according to a previous study (5). However, this treatment regimen has not been a consensus in many countries due to potential complications and uncertain survival benefits. In particular, the debate about HIPEC became more intense after the results of the PRODIGE 7 trial were published. The PRODIGE 7 trial showed no evidence of additional survival benefits with CRS plus HIPEC compared with CRS alone for colorectal peritoneal metastases (8). Of note, the PRODIGE study did not distinguish between metachronous and synchronous colorectal peritoneal metastases. Metachronous colorectal peritoneal metastases were associated with early recurrence after CRS with HIPEC compared with synchronous colorectal peritoneal metastases (9). Besides, 30 min HIPEC with high­dose oxaliplatin seems not appropriate in the PRODIGE 7 trial.

Based on the above controversy, this retrospective, observational study aimed to analyze whether CRC patients with synchronous peritoneal metastases could benefit from HIPEC after up-front CRS at a tertiary medical center in China.

## Methods

### Patient selection

**Figure 1:**
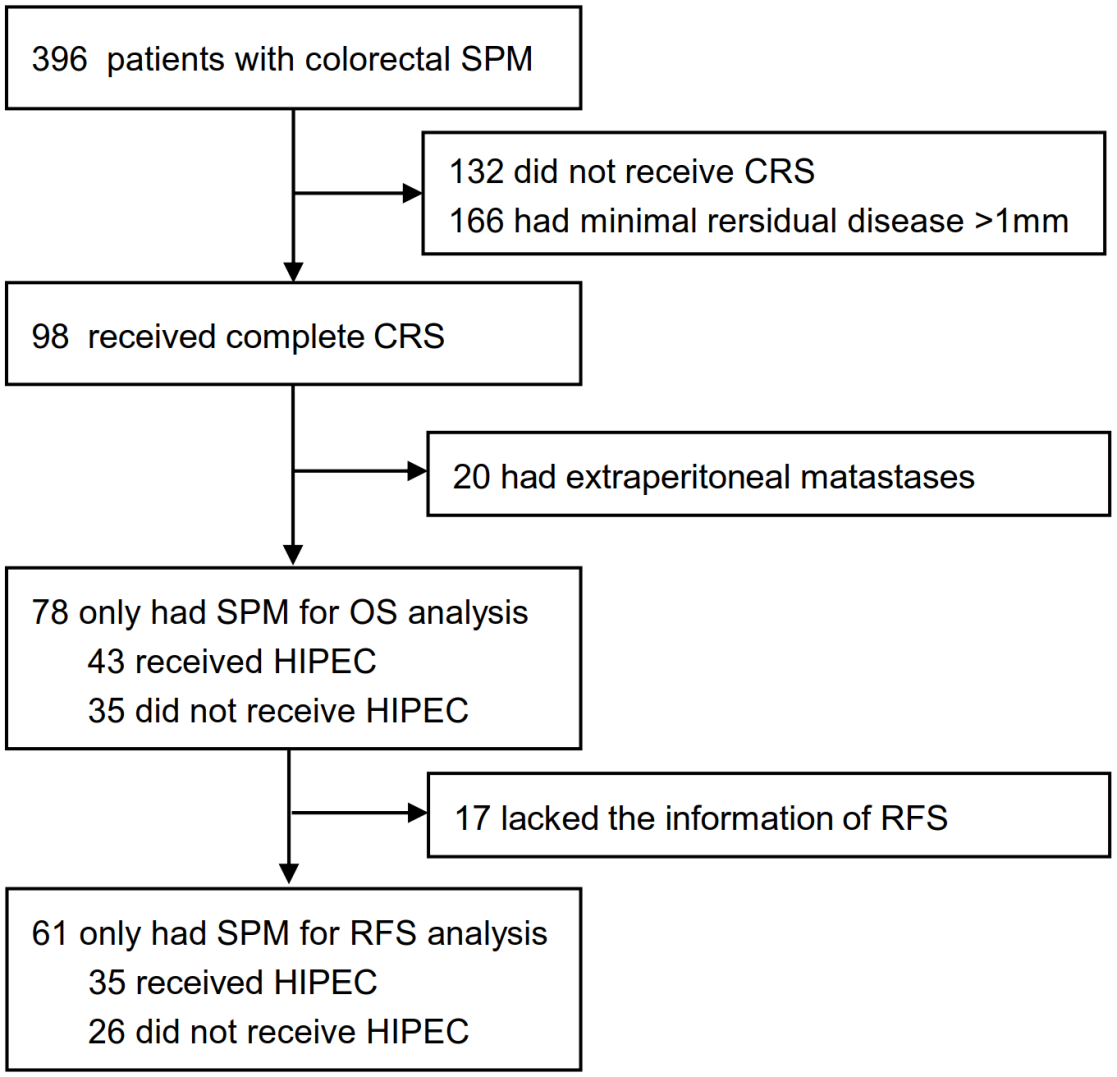
Patients selectionand treatment allocation. HIPEC, hyperthermic intraperitoneal chemotherapy; CRS, cytoreductive Surgery; SPM, synchronous peritoneal metastases; OS, overall survival; RFS, relapse-free survival.

Patients diagnosed with synchronous colorectal peritoneal metastases in the Sixth Affiliated Hospital of Sun Yat-sen University, Guangzhou, China, were initially considered from September 1, 2012, to September 1, 2019. Patients were excluded in cases of incomplete CRS, concomitant extraperitoneal metastases, or incomplete clinicopathological data. According to whether patients received HIPEC after CRS, the enrolled patients were divided into CRS plus HIPEC group and CRS alone group (**Figure 1**). All the data were from the Database of Colorectal Cancer in our hospital. The study was approved by the Ethics Committee of the Sixth Affiliated Hospital of Sun Yat-sen University (No. 2020ZSLYEC–109).

### Cytoreductive surgery

CRS involved removal of the primary tumor and the invaded organs, dissection of lymph nodes, and peritonectomy. CRS was usually performed after evaluation by a multidisciplinary team. The goal of CRS was to remove all macroscopic diseases without residual disease. If macroscopically complete cytoreduction was challenging to achieve, partial cytoreduction was selected to relieve the patient’s symptoms, or the abdominal cavity was closed only after the biopsy. The extent of peritoneal metastases was assessed by intraoperative exploration and scored using peritoneal cancer index (PCI) (10, 11). The completeness of cancer resection (CCR) was evaluated at the end of the procedure (10).

### HIPEC procedure

In China, the prophylactic application of HIPEC has not been recognized by all colorectal surgeons. Therefore, some patients who received complete CRS didn’t undergo HIPEC. In this study, HIPEC was performed postoperatively using a closed technique. 5-Fluorouracil (5-FU) and oxaliplatin are effective systemic chemotherapy agents for colorectal cancer. Oxaliplatin needs to be dissolved in a glucose solution, which may cause hyperglycemia and severe disturbances in electrolyte concentrations (12, 13). Over the past decade, 5-FU was the first choice for HIPEC in CRC patients, according to the Chinese expert consensus.

More specifically, HIPEC was carried out in the following way: (1) At the end of CRS, four perfusion tubes were placed in the upper abdominal hepatorenal recess, splenic hilum, and pelvic floor on both sides through the puncture holes on both sides of the abdominal wall and interlaced internally. (2) Within 1-3 days after surgery, HIPEC was performed in the ward. Generally, prophylactic pethidine and promethazine were used for sedation and analgesia. The perfusion tubes were connected to the peritoneal thermal perfusion treatment system. The perfusion fluid was injected into and discharged from the peritoneal cavity at a constant temperature, speed, and quantity. (3) Specific parameters. The dose of 5-FU usually was 600mg/m^2^. The perfusion fluid was usually 3-4L normal saline, and the principle was to fill the abdominal cavity and circulate normally. The temperature of the perfusion fluid was 42°C. The perfusion rate was 400-600mL per minute. Perfusion time was 60 min. In general, HIPEC was performed 2-3 times after surgery.

### Covariates

Covariates included sex, age, carcinoembryonic antigen (CEA) level, carbohydrate antigen 19-9 (CA19-9) level, carbohydrate antigen 125 (CA125) level, tumor histology, histological differentiation, tumor location, T stage, lymph node metastasis, and extraperitoneal metastases at diagnosis. Systemic chemotherapy included preoperative and postoperative chemotherapy, and they were carried out under the guidance of oncologists. PCI was recorded according to the results of intraoperative exploration. All tumors were staged according to the 8th edition of the American Joint Committee on Cancer (AJCC) TNM staging system.

### Primary outcomes

Overall survival (OS) was defined as the time from primary treatment until death due to any cause or the date of the last follow-up in censored patients. Relapse-free survival (RFS) was defined as the time between primary treatment and the first distant or peritoneal relapse. All patients were followed at 3-month intervals during the first two years, at least every six months after that for an additional period of 3 years, and then once a year.

### Statistical analysis

In this study, continuous variables were presented as the median with range and compared with the Mann-Whitney U test if they didn’t coincide with normal distribution. Categorical data were presented as proportions and compared using the *χ*2 test or Fisher’s exact test. Univariate and multivariate COX proportional hazard regression analyses were conducted to identify prognostic factors. Covariates with *P* < 0.05 by univariate analysis were subjected to multivariate analysis. Kaplan–Meier method and log-rank test were performed to estimate differences in overall survival and relapse-free survival. A two-sided *P* < 0.05 was considered statistically significant. All statistical analyses were performed with R software (Version 4.0.3).

## Results

Between Sep 1, 2012, and Sep 1, 2019, 396 patients with synchronous colorectal peritoneal metastases were initially analyzed, and 78 patients were identified for this study. Complete overall survival information was available for the 78 patients, 43 of whom received HIPEC, and 35 of whom did not. In this study, 38 received 5-FU-based HIPEC, 3 received 5-FU and oxaliplatin combined regimen, and 2 received oxaliplatin monotherapy regimen. Seventeen patients had incomplete relapse-free survival (RFS) information, so only 61 patients were used for relapse-free survival (RFS) analysis (**Figure 1**). The demographic characteristics of the CRS plus HIPEC group and CRS group were similar (**Table 1** **;** **Supplementary Table S1**). All 78 patients had no prior surgical history. Eight patients (10.3%) received preoperative neoadjuvant therapy, and fifty patients (19.2%) received postoperative adjuvant systemic chemotherapy in 78 patients (**Supplementary Table S2**). The PCI scores were less than 12 in all patients, with a median PCI of 4 (IQR, 2-6), which suggested that these patients had relatively low-volume peritoneal metastases.

**Table 1:** Baseline characteristics in 78 patients.

Characteristics	CRS plus HIPEC	CRS group	*P*
	group		
**Age**			0.496
≤ 60	29 (67.4)	21 (60.0)	
> 60	14 (32.6)	14 (40.0)	
**Sex**			0.235
Male	29 (67.4)	19 (54.3)	
Female	14 (32.6)	16 (45.7)	
**CEA (ng/ml)**			0.331
≤ 10	28 (65.1)	19 (54.3)	
> 10	15 (34.9)	16 (45.7)	
**CA19-9 (U/ml)**			0.299
≤ 37	35 (81.4)	25 (71.4)	
> 37	8 (18.6)	10 (28.6)	
**CA125 (U/ml)**			0.836
≤ 35	28 (65.1)	22 (62.9)	
> 35	15 (34.9)	13 (37.1)	
**Tumor histology**			
Adenocarcinoma	33 (76.7)	23 (65.7)	0.367
Mucinous adenocarcinoma	10 (23.3)	11 (31.4)	
Signet-ring cell carcinoma	0 (0)	1 (2.9)	
**Histological differentiation**			0.583
Low or undifferentiated	17 (39.5)	.16 (45.7)	
High or moderate	26 (60.5)	19 (54.3)	
**Tumor location**			0.214
Left-sided colon	15 (34.9)	17 (48.6)	
Right-sided colon	22 (51.2)	11 (31.4)	
Rectum	6 (14.0)	7 (20.0)	
**T stage**			0.027
T1-3	15 (34.9)	21 (60.0)	
T4	28 (65.1)	14 (40.0)	
**Lymph node metastasis**			0.506
Negative	10 (23.3)	6 (17.1)	
Positive	33 (76.7)	29 (82.9)	
**Preoperative systemic chemotherapy**			0.758
No	39 (90.7)	31 (88.6)	
Yes	4 (9.3)	4 (11.4)	
**Adjuvant systemic chemotherapy**			0.836
No	15 (34.9)	13 (37.1)	
Yes	28 (65.1)	22 (62.9)	
**PCI, median (IQR)**	4 (2-6)	4 (2-5)	0.028

PCI, peritoneal cancer index; IQR, interquartile range.

In all 78 patients, the median follow-up time was 46.0 months, and 35 patients (44.9%) died. Corresponding numbers were 46.0 (95% CI, 34.2-57.8) months and 12 deaths (34.3%) in the CRS plus HIPEC group and 46.0 (95% CI, 24.9-67.1) months and 23 (65.7%) deaths in the CRS alone group. The median overall survival was 63 months, and the 3-year and 5-year overall survival (OS) rates were 58.8% and 50.9% for all the patients. For 61 patients with recurrence data, the median follow-up time of patients was 49.0 months, and 40 patients (65.6%) had a relapse or died. The corresponding numbers were 47.0 (95% CI, 25.6-68.4) months and 20 (57.1%) had a relapse or died in the CRS plus HIPEC group and 54.0 (95% CI, 15.6-92.4) months and 20 (76.9%) had a relapse or died in the CRS alone group. The median relapse-free survival was 20.0 (95% CI, 12.3-27.7) months, and the 3-year and 5-year relapse-free survival rates were 36.8% and 29.1% for the 61 patients.

**Figure 2:**
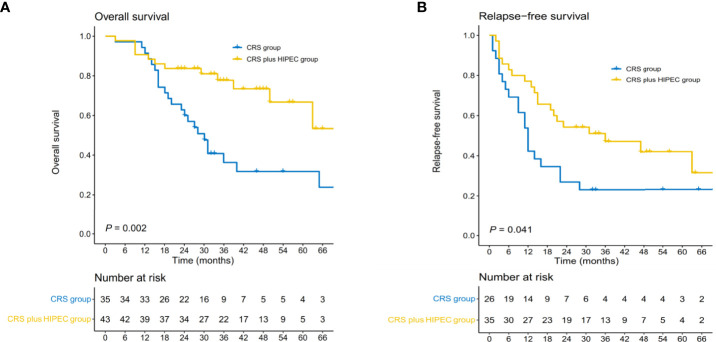
Kaplan-Meier estimates of overall survival **(A)** and relapse-free survival **(B)**.

HIPEC significantly increased survival benefits for the entire population. After a median follow-up of 46.0 months, 5-year overall survival was 66.8% and the median relapse-free survival was 36.0 (95% CI, 6.8-65.1) months in the CRS plus HIPEC group. 5-year overall survival was 31.2% and the median relapse-free survival was 12.0 (95% CI, 9.0-15.0) months in the CRS alone group. (**Figure 2**).

The association between clinicopathological factors and outcomes was investigated using univariate and multivariate Cox proportional hazard regression analyses; HIPEC was an independent prognostic factor for overall survival (*P* = 0.004) and relapse-free survival (*P* = 0.049) (**Tables 2** **,** **3**).

**Table 2:** Univariate and multivariate analyses for OS of CRC patients with isolated synchronous colorectal peritoneal metastases using the Cox proportional hazards regression.

	Univariate analysis		Multivariate analysis	
Variable	HR (95%CI)	*P* value	HR (95%CI)	*P* value
**Age (>60 years)**	1.639 (0.825-3.254)	0.158		
**Sex (Female)**	0.748 (0.369-1.515)	0.420		
**CEA (>10 ng/mL)**	1.016 (0.516-1.998)	0.964		
**CA19-9 (>37 U/mL)**	1.743 (0.801-3.793)	0.161		
**CA125 (> 35 U/mL)**	1.379 (0.700-2.715)	0.353		
**Tumor histology**		0.026		0.757
Adenocarcinoma	Ref		Ref	
Mucinous adenocarcinoma	2.285 (1.114-4.686)	0.024	1.170 (0.405-3.378)	0.772
Signet-ring cell carcinoma	6.590 (0.847- 51.275)	0.072	2.309 (0.256-20.863)	0.456
**Histological differentiation**				
Low or undifferentiated	Ref		Ref	
High or moderate	0.431 (0.217-0.858)	0.016	0.472 (0.168-1.329)	0.472
**Tumor location**		0.152		
Right-sided colon	Ref			
Left-sided colon	0.473 (0.222-1.008)	0.052		
Rectum	0.715 (0.281-1.822)	0.537		
**T stage**				
T1-3	Ref			
T4	1.220 (0.617-2.414)	0.567		
**Lymph node metastasis (Yes)**	1.327 (0.550-3.198)	0.529		
**Preoperative systemic chemotherapy (Yes)**	0.737 (0.225-2.416)	0.614		
**Adjuvant systemic chemotherapy (Yes)**	0.855 (0.435-1.683)	0.651		
**HIPEC (Yes)**	0.347 (0.172-0.702)	0.003	0.349 (0.170-0.718)	0.004

OS, overall survival; HIPEC, hyperthermic intraperitoneal chemotherapy.

**Table 3:** Univariate and multivariate analyses for RFS of CRC patients with isolated synchronous colorectal peritoneal metastases using the Cox proportional hazards regression.

	Univariate analysis		Multivariate analysis	
Variable	HR (95%CI)	*P* value	HR (95%CI)	*P* value
**Age (>60 years)**	1.162 (0.598-2.258)	0.658		
**Sex (Female)**	0.434 (0.211-0.894)	0.023	0.440 (0.210-0.921)	0.029
**CEA (>10 ng/mL)**	0.763 (0.402-1.450)	0.410		
**CA19-9 (>37 U/mL)**	1.506 (0.730-3.106)	0.268		
**CA125 (>35 U/mL)**	1.659 (0.883-3.118)	0.116		
**Tumor histology**		0.004		0.380
Adenocarcinoma	Ref		Ref	
Mucinous adenocarcinoma	2.604 (1.350-5.021)		1.592 (0.563-4.500)	
**Histological differentiation**		0.010		0.483
Low or undifferentiated	Ref		Ref	
High or moderate	0.440 (0.235-0.825)		0.701 (0.260-1.889)	
**Tumor location**		0.180		
Right-sided colon	Ref			
Left-sided colon	0.518 (0.257-1.045)	0.066		
Rectum	0.667 (0.264-1.685)	0.392		
**T stage**				
T1-3	Ref			
T4	1.440 (0.768-2.703)	0.256		
**Lymph node metastasis (Yes)**	1.751 (0.733-4.182)	0.208		
**Preoperative systemic chemotherapy (Yes)**	1.494 (0.581-3.837)	0.405		
**Adjuvant systemic chemotherapy (Yes)**	1.121 (0.570-2.207)	0.740		
**HIPEC (Yes)**	0.501 (0.266-0.944)	0.047	0.527 (0.278-0.996)	0.049

RFS, relapse-free survival; HIPEC, hyperthermic intraperitoneal chemotherapy.

No patients died within 30 days in the CRS plus HIPEC group or CRS alone group. No grade III or higher complications in the CRS alone group. However, in the CRS plus HIPEC group, 5 patients had complications of grade IIIa, including anastomotic leakage, intestinal obstruction, and gastroparesis (**Supplementary Table S3**). These complications were improved by conservative treatment.

## Discussion

In this retrospective study, the addition of HIPEC following up-front CRS improved overall survival and relapse-free survival in colorectal cancer patients with isolated synchronous peritoneal metastases. Findings of this study were different from the controversial PRODIGE 7 study. Inconsistent results may enable us to rethink the indications of HIPEC and prompt us to conduct randomized trials.

The present study appears to be similar to the PRODIGE 7 study, but it is fundamentally different (8). First of all, the patients enrolled were all patients with synchronous colorectal peritoneal metastases in the present study, while most of those in the PRODIGE 7 study had prior surgery. Secondly, the most significant difference lies in the different regimens of HIPEC. Patients received HIPEC with 5-FU, which usually lasted 60min and was performed 2-3 times after surgery in this study, while 30 min oxaliplatin-based HIPEC was conducted in the trial of PRODIGE 7. Thirdly, in terms of systemic chemotherapy, patients received higher proportions of targeted therapy (72% in the CRS plus HIPEC group; 73% in the CRS alone group) and oxaliplatin-based systemic chemotherapy in the PRODIGE 7 trial. On the other hand, although some previous retrospective studies have explored the role of complete CRS combined with or without HIPEC on survival (14, 15). There is no retrospective study to compare the additional benefit of HIPEC. Therefore, although the present study is a single-center, retrospective study, it has evident innovation.

In the CRS alone group, the median overall survival (OS) was 30 months, lower than the 41 months reported in the PRODIGE 7 study. The inferior median overall survival (OS) may be owing to several reasons. Firstly, using a higher proportion of targeted agents and postoperative adjuvant chemotherapy in the PRODIGE 7 study improved patient outcomes. Previous studies have shown that targeted drugs can improve outcomes in patients with advanced colorectal cancer (16–18). Ceelen et al. reported that patients receiving HIPEC had a better prognosis with preoperative intravenous bevacizumab compared with those without bevacizumab (median overall survival 39 vs 22 months) (17). In addition, almost all of the patients in the PRODIGE 7 study received systemic chemotherapy after surgery, whereas most of the patients at our center did not receive postoperative chemotherapy. The main reasons may be that bevacizumab and cetuximab were approved in Mainland China late, and most families could not afford the high cost of chemotherapy drugs.

Median relapse-free survival (RFS) in the CRS alone group was similar to that in the PRODIGE 7 study (12.0 vs 11.1 months). However, the relapse-free survival of the CRS plus HIPEC group was significantly better than that of the PRODIGE 7 study (30.0 vs 13.1 months). The main reason might be that the HIPEC technique in the present study can effectively remove minimal residual disease and reduce the recurrence. Compared to the PRODIGE 7 study, longer perfusion time may bring more thorough clearance of residual tumor cells or lesions, resulting in better relapse-free survival (RFS) in patients receiving CRS plus HIPEC. Moreover, it is well known that PCI is one of the most powerful prognostic factors for survival after CRS with HIPEC (19–21). In the present study, the median PCI was 4, much lower than the previous studies (8, 21). This may be an important reason why patients have better relapse-free survival in the CRS plus HIPEC group. Low PCI may be one of the best indications for HIPEC after CRS for synchronous colorectal peritoneal metastases. On the other hand, preoperative chemotherapy with FOLFOX may induce oxaliplatin resistance and reduce the efficacy of postoperative chemotherapy, while more than 40% of patients in the PRODIGE 7 study received oxaliplatin-based preoperative chemotherapy (22).

Of note, HIPEC with 5-FU alone was rarely reported in the world (23). Several studies have shown that 5-FU had no synergistic effect with hyperthermia and required prolonged exposure to induce cancer cell death (24–26). However, as one of the systemic chemotherapy drugs, the killing effect on tumor cells of 5-FU is beyond doubt (26). In this study, 5-FU-based postoperative HIPEC could reduce chemotherapy toxicity and had a lower incidence of postoperative complications. In addition, for most developing countries, the price of 5-FU is relatively low. Combined with domestic equipment, 5-FU-based postoperative HIPEC can reduce treatment costs (27).

The efficacy of HIPEC is controversial. In addition to PRODIGE 7, two randomized controlled clinical trials suggested that oxaliplatin-based prophylactic HIPEC could not significantly reduce the occurrence of colorectal peritoneal metastases (28, 29). The results of the COLOPEC trial revealed that oxaliplatin-based adjuvant HIPEC didn’t improve peritoneal relapse-free survival at 18 months for patients with T4 and perforated colon cancer (28). However, the COLOPEC trial has several limitations. Firstly, this study did not include patients with synchronous colorectal peritoneal metastases who received complete CRS. Previous studies have identified synchronous colorectal peritoneal metastases as a risk factor for metachronous peritoneal metastases and may be the highest risk factor. Secondly, in the COLOPEC study, 79 patients (79/87, 91%) received adjuvant HIPEC 5-8 weeks after primary tumor resection. According to studies in basic oncology, surgery can adversely affect the peritoneal ecosystem. Most growth factors, chemokines, and cytokines that promote wound healing can also promote tumor growth, invasion, and angiogenesis. Besides, abdominal adhesion has not been formed in the early postoperative period, which facilitates the distribution of chemotherapy fluid. When HIPEC treatment was performed 5-8 weeks after radical resection, 66% of patients had experienced intraperitoneal adhesions in different degrees. Even if adhesiolysis was completed, the actual efficacy of HIPEC might be reduced to a certain extent. Hence, early postoperative application of HIPEC may be most effective. Finally, the 30min oxaliplatin-based HIPEC is also controversial for the short time. Therefore, the negative results of the COLOPEC study seem reasonable, and the efficacy of prophylactic HIPEC cannot be denied completely. Compared with the COLOPEC study, the PROPHYLOCHIP–PRODIGE 15 trial included patients with synchronous or localized peritoneal metastases, except for patients with T4 and perforation (29). However, it was designed to compare the survival benefit of systemic secondary exploration plus HIPEC versus standard surveillance and did not intend to identify the potential benefit of prophylactic HIPEC. In the second-look surgery group, about half of the 71 (37/71) patients had colorectal peritoneal metastases when performing peritoneal exploration, suggesting that HIPEC should be implemented early after primary surgery.

In terms of complications, the CRS plus HIPEC group had more grade III postoperative complications than the CRS alone group in this study. However, the overall complication rate was lower than previously reported (8, 30), which may be related to surgical proficiency and lower PCI. On the other hand, the patients enrolled in this study had a low degree of peritoneal metastases, leading to relatively limited surgical resection. Therefore, CRS plus HIPEC can be performed in experienced centers for appropriate patients.

### Limitations

There are some limitations of this study. Firstly, the number of patients is a major limitation of this study. Furthermore, fewer patients were analyzed for relapse-free survival (RFS) after excluding patients with unclear relapse status. However, after a relatively strict patient selection, the baseline data of the two groups were matched. Therefore, the present study was reliable. Certainly, rigorous randomized controlled trials are needed in the future. Secondly, the intraperitoneal chemotherapeutic drug in this study was 5-FU, while platinum-based cytotoxic drugs and mitomycin were more likely to be selected in most countries (23, 31). From the current point of view, 5-FU may not be the optimal choice. Therefore, the regimen of HIPEC in this study limited the extensibility. However, the better survival benefit in the present study may help us regain confidence in HIPEC and provide essential references for future clinical trials.

## Conclusions

In conclusion, the present study suggests that the addition of HIPEC following up-front CRS improved overall survival and relapse-free survival in colorectal cancer patients with isolated synchronous peritoneal metastases. Certainly, rigorous randomized controlled trials are needed in the future.

## Data availability statement

The raw data of the present study is available from the corresponding authors with a reasonable request.

## Ethics statement

The studies involving human participants were reviewed and approved by the Ethics Committee of the Sixth Affiliated Hospital, Sun Yat-sen University. Written informed consent for participation was not required for this study in accordance with the national legislation and the institutional requirements.

## Author contributions

XQ, LY, HuW and HuaW contributed to the conception and design of the study. XQ, MM, YZ, YC, ZW and RL contributed to data acquisition. XQ, MM, YZ performed the statistical analysis. XQ wrote the first draft of the manuscript. MM and YZ wrote sections of the manuscript. All authors contributed to manuscript revision, read, and approved the submitted version.

## Funding

This study was funded by the National Natural Science Foundation of China (Grant No. 82103084) and the Sun Yat-sen University Clinical Research 5010 Program (Grant No. 2019021).

## Conflict of interest

The authors declare that the research was conducted in the absence of any commercial or financial relationships that could be construed as a potential conflict of interest.

## Publisher’s note

All claims expressed in this article are solely those of the authors and do not necessarily represent those of their affiliated organizations, or those of the publisher, the editors and the reviewers. Any product that may be evaluated in this article, or claim that may be made by its manufacturer, is not guaranteed or endorsed by the publisher.
