# Supplementary material for: Hyperthermic intraperitoneal chemotherapy following up-front cytoreductive surgery versus cytoreductive surgery alone for isolated synchronous colorectal peritoneal metastases: A retrospective, observational study
Source: Front Oncol. 2022 Oct 18;12:959514. doi: 10.3389/fonc.2022.959514 (PMC9623104; doi:10.3389/fonc.2022.959514)
Supplement: Supplementary file 1 [file DataSheet_1.pdf]

Supplementary Table S1 Baseline characteristics in 61 patients for RFS analysis

**Characteristics**	**CRS plus HIPEC group**	**CRS group**	***P***
**Age**			0.793
≤ 60	24 (68.6)	17 (65.4)	
> 60	11 (31.4)	9 (34.6)	
**Sex**			0.382
Male	24 (68.6)	15 (57.7)	
Female	11 (31.4)	11 (42.3)	
**CEA (ng/ml)**			0.856
≤ 10	21 (60.0)	15 (57.7)	
> 10	14 (40.0)	11 (42.3)	
**CA19-9 (U/ml)**			0.356
≤ 37	29 (82.9)	19 (73.1)	
> 37	6 (17.1)	7 (26.9)	
**CA125 (U/ml)**			0.839
≤ 35	22 (62.9)	17 (65.4)	
> 35	13 (37.1)	9 (34.6)	
**Tumor histology**			
Adenocarcinoma	27 (77.1)	18 (69.2)	0.487
Mucinous adenocarcinoma	8 (22.9)	8 (30.8)	
**Histological differentiation**			0.523
Low or undifferentiated	12 (34.3)	11 (42.3)	
High or moderate	23 (65.7)	15 (57.7)	
**Tumor location**			0.394
Left-sided colon	12 (34.9)	13 (50.0)	
Right-sided colon	18 (51.4)	9 (34.6)	
Rectum	5 (14.3)	4 (15.4)	
**T stage**			0.171
T1-3	14 (40.0)	15 (57.7)	
T4	21 (60.0)	11 (42.3)	
**Lymph node metastasis**			0.940
Negative	7 (20.0)	5 (19.2)	
Positive	28 (80.0)	21 (80.8)	
**Preoperative systemic chemotherapy**			0.446
No	32 (91.4)	22 (84.6)	

Yes	3 (8.6)	4 (15.4)	
**Adjuvant systemic chemotherapy**			0.956
No	11 (31.4)	8 (30.8)	
Yes	24 (68.6)	18 (69.2)	
**PCI, median (IQR)**	4 (2-6)	4 (2-6)	0.147

PCI, peritoneal cancer index; IQR, interquartile range.

Supplementary Table S2 Systemic chemotherapy regimens in patients who received preoperative and postoperative chemotherapy in 78 patients

Chemotherapy regimens	CRS plus HIPEC	CRS alone
**Preoperative systemic chemotherapy regimens**	n=4	n=4
**FOLFOX**	2	3
**FOLFOX + bevacizumab**	1	0
**FOLFOXIRI**	1	0
**FOLFOXIRI+ bevacizumab**	0	1
**Postoperative systemic chemotherapy regimens**	n=28	n=22
**FOLFOX**	18	17
**XELOX**	1	2
**FOLFOX + bevacizumab**	4	0
**FOLFOX + cetuximab**	1	0
**FOLFIRI**	1	1
**FOLFIRI + bevacizumab**	0	2
**Cetuximab**	2	0
**Cetuximab + Capecitabine**	1	0

Supplementary Table S3 Details of Clavien-Dindo grade ≥ 3 postoperative morbidity at 30 days

Adverse event	CRS plus HIPEC	CRS alone
**Anastomotic leakage, grade 3a**	3	0
**Ileus, grade 3a**	1	0
**Gastroparesis, grade 3a**	1	0
